# A Comparative Study of Hemorrhagic Conversion Patterns After Stroke Thrombolysis With Alteplase Versus Tenecteplase

**DOI:** 10.7759/cureus.46889

**Published:** 2023-10-12

**Authors:** Mohamad Ezzeldin, Courtney Hill, Ali Kerro, Eryn Percenti, Adam Delora, Juan Santos, Hamzah Saei, Lisa Greco, Rime Ezzeldin, Mohammad El-Ghanem, Yazan Alderazi, Yana Kim, Cathleen Poitevint, Osman Mir

**Affiliations:** 1 Clinical Sciences, University of Houston, Houston, USA; 2 Emergency Medicine, Hospital Corporation of America (HCA) Houston Healthcare Kingwood, Houston, USA; 3 Neurology, Hospital Corporation of America (HCA) Houston Healthcare Conroe, Conroe, USA; 4 Internal Medicine, Hospital Corporation of America (HCA) Houston Healthcare Kingwood, Houston, USA; 5 Neurology, Corpus Christi Medical Center, Corpus Chrsiti, USA; 6 Neurology, Rio Grande Regional Hospital, McAllen, USA; 7 Neurology, Valley Baptist Medical Center, Harlingen, USA; 8 Neurology, Hospital Corporation of America (HCA) Houston Healthcare Gulf Coast Division, Houston, USA; 9 Medicine, Jordan University of Science and Technology, Irbid, JOR; 10 Neurology, Hospital Corporation of America (HCA) Houston Healthcare Northwest, Houston, USA; 11 Neuroendovascular Surgery, Hospital Corporation of America (HCA) Houston Healthcare Clear Lake, Houston, USA; 12 Neurology, Texas Stroke Institute, Plano, USA

**Keywords:** tenecteplase (tnk), alteplase (rt-pa), hemorrhagic conversion, stroke thrombolysis, stroke

## Abstract

Background and purpose: Tenecteplase is the thrombolytic drug of choice for acute ischemic stroke (AIS) as it has unique pharmacologic properties, along with results demonstrating its non-inferiority compared to alteplase. However, there are contradictory data concerning the risk of intracranial hemorrhage. The purpose of the study was to report the rate and patterns of symptomatic intracranial hemorrhage (sICH) in AIS patients after thrombolysis with tenecteplase compared to alteplase.

Methods: This is a retrospective cohort study with data collected 90 days before and after the change from alteplase to tenecteplase from 15 Texas stroke centers. The primary endpoint is the incidence of sICH according to the Safe Implementation of Thrombolysis in Stroke-Monitoring Study (SITS-MOST) and European Cooperative Acute Stroke Study III (ECASS-3) criteria. The secondary endpoints are the radiographic pattern of hemorrhagic conversion according to the Heidelberg bleeding classification (HBC).

Results: A total of 431 patients were eligible for thrombolytic therapy. Half of the cohort received alteplase (n=216), and the other half received tenecteplase (n=215). The average age of the alteplase group was 62.94 years old (SD=15.12) and 64.45 years old (SD=14.51) for the tenecteplase group. Seven patients in the alteplase group (3.2%) and 14 (6.5%) in the tenecteplase group had sICH, with an odds ratio of 1.44 (95% CI 0.60-3.43; P=0.41). An increased National Institutes of Health Stroke Scale (NIHSS) score on arrival (1.06; 95% CI 1.0004-1.131; P=0.04) was a statistically significant predictor of sICH. Tenecteplase was associated with a statistically significant increase in HBC-3 (P=0.040) over alteplase.

Conclusions: Compared with alteplase, our study revealed a higher rate of sICH with tenecteplase that was not statistically significant and a higher rate of HBC-3 hemorrhages that was statistically significant. The proposed mechanism of bleeding is hemorrhagic conversion in clinically silent infarcts and contusions underlying the lesions. Further studies are needed to confirm our findings and determine predictive risk factors.

## Introduction

Alteplase was approved by The United States Food and Drug Administration (FDA) in 1996 to treat acute ischemic stroke (AIS) [1]. Recombinant tissue plasminogen activator (rt-PA) is the standard treatment for AIS due to its ability to improve neurological outcomes when administered within 4.5 hours from symptom onset [2,3]. Despite its efficacy, one of the factors limiting the use of alteplase is the risk of symptomatic intracranial hemorrhage (sICH). The risk of sICH reported in the literature varies between 2% and 6% [4].

Tenecteplase has been used instead of alteplase as the thrombolytic agent of choice for AIS across different healthcare systems. Tenecteplase is a genetically modified variant of alteplase obtained through recombinant DNA technology [5]. Randomized trials showed non-inferiority of tenecteplase compared to alteplase in AIS patients, with a similar safety profile when using a 0.25 mg/kg (maximum 25 mg) dose rather than 0.4 mg/kg (maximum 40 mg) [6-11]. This is in contrast to the cardiac dosing of 0.53 mg/kg (maximum 50 mg) [5].

A literature review demonstrates comparable safety between tenecteplase and alteplase [10-14]. Tenecteplase administration offers several advantages over alteplase. Its high fibrin specificity makes it more targeted in its action. Additionally, tenecteplase is administered as a single bolus dose, which is believed to improve administration efficiency and reduce transfer times [15-18]. Tenecteplase can be associated with improved functional outcomes and a higher recanalization rate than alteplase in patients with a confirmed large vessel occlusion [19]. It has also been shown to be cost-effective [20]. In 2019, the American Heart Association (AHA)/American Stroke Association guidelines were updated to include considering the use of tenecteplase over alteplase in eligible patients, including tenecteplase as a single 0.25 mg/kg bolus (maximum 25mg) before mechanical thrombectomy or as a single bolus of 0.4 mg/kg in patients with minor neurological impairment and no major intracranial occlusion (class IIb recommendation) [21]. Similarly, the European Stroke Organization and the Australian and New Zealand clinical guidelines for acute stroke management include tenecteplase as a potential thrombolytic agent for treating acute ischemic stroke [22,23]. 

While tenecteplase has several advantages and has shown non-inferiority compared to alteplase, it has not yet been approved by the FDA for the treatment of AIS. A large study comparing the safety of tenecteplase to alteplase for AIS found that higher rates of sICH were seen [24]. In contrast, a recently published study reported lower rates of sICH with tenecteplase than alteplase [25].

This study aims to investigate the rate of sICH as well as the patterns of hemorrhagic conversions associated with tenecteplase while using alteplase as a comparison. To our knowledge, this is the first article to describe hemorrhagic patterns with tenecteplase across multiple different stroke centers.

## Materials and methods

Patient sample and study design

This is a retrospective cohort study across 15 stroke centers in Texas, with incorporated data from 10 primary and five comprehensive stroke centers across the Hospital Corporation of America (HCA) Gulf Coast and HCA North Texas Divisions. Patient data were collected if the following inclusion criteria were met: age 18 years or older, suspected to have an AIS, were eligible for thrombolytic therapy and received either IV tenecteplase or alteplase at the standard dose. Any patients who met the above criteria were included in the study if they received a thrombolytic within the 90 days before and the 90 days after the stroke center transitioned from alteplase to tenecteplase. Hence, three months of alteplase and three months of tenecteplase-administered stroke population data were included. Exclusion criteria included pregnancy, prior intracranial hemorrhage, and patients who had any contraindications to thrombolytic therapy or did not meet the criteria to receive thrombolytic therapy. The alteplase population received the standard dose of 0.9 mg/kg (not to exceed 90 mg total treatment dose). The tenecteplase population received 0.25 mg/kg (not to exceed 25 mg total treatment dose). Patients were prospectively identified as having sICH based on the Safe Implementation of Thrombolysis in Stroke-Monitoring Study (SITS-MOST) and European Cooperative Acute Stroke Study III (ECASS-3) criteria as part of stroke center quality metrics reporting. Four fellowship-trained neuroendovascular or vascular neurologists reviewed the imaging for all hemorrhagic conversion cases and independently graded them according to the Heidelberg bleeding classification (HBC) [26]. Disagreements in grading were settled by consensus.

A primary stroke center was defined as a hospital that can offer services and resources to patients with acute stroke, including intravenous thrombolytics. In contrast, a comprehensive stroke center includes hospitals that can provide the above services in addition to neurosurgical, neurocritical, and neuroendovascular therapies.

Outcome measures

The primary endpoint was to compare the incidence of sICH according to SITS-MOST/ECASS-3 criteria in the alteplase and tenecteplase groups [3,27]. The secondary endpoint included the radiographic pattern of hemorrhagic conversion according to the HBC [26]. Heidelberg bleeding classification grade 2 is defined as a hematoma occupying 30% or more of the infarcted tissue, with an obvious mass effect representing parenchymal hematoma (PH2). Heidelberg bleeding classification grade 3 is intracerebral hemorrhage outside the infarcted brain tissue or intracranial-extracerebral hemorrhage, representing remote parenchymal hematoma (RPH). Both HBC 2 and HBC 3 reflect severe hemorrhage [28].

Statistical analyses

The data were analyzed in Python using the statistics model 0.13.5. The variance inflation factor was computed to look for multicollinearity among the independent variables [29]. A binary logistic regression was performed on the dependent variable, the presence or absence of sICH. Independent variables included gender and history of hyperlipidemia, smoking, prior stroke, atrial fibrillation, coronary artery disease, if mechanical thrombectomy was attempted, and whether the patient received alteplase or tenecteplase. Smoking was excluded from the analysis because most of the population were non-smokers.

## Results

A total of 431 patients were included in this study based on the above eligibility criteria. There were 305 patients abstracted from the HCA Gulf Coast Division and 126 from the HCA North Texas Division. Half of the cohort received alteplase (n=216), and the other half received tenecteplase (n=215). Females comprised 51% (n=110) of the alteplase cohort and 54% (n=117) of the tenecteplase cohort. The average age of the alteplase group was 62.94 (SD=15.12) and 64.45 (SD=14.51) for the tenecteplase group. Almost two-thirds of the study population reported no smoking history: 66% for alteplase and 64% for tenecteplase. Most patients received thrombolytic therapy within three hours of symptom onset: 174 (81%) for alteplase versus 176 (82%) for tenecteplase. Endovascular thrombectomy was attempted on 34 patients (15%) in the alteplase group, compared to 26 patients (12%) in the tenecteplase group. As seen in Table 1, there is no statistically significant difference in population demographics between the cohorts.

**Table 1:** Characteristics of the study population NIHSS: National Institutes of Health Stroke Scale

Clinical values	Alteplase (N=216)	Tenecteplase (N=215)	P-value
Gender, No. (%)			0.53
Male	106 (49.07)	98 (45.58)	
Female	110 (50.93)	117 (54.52)	
Age, mean (SD)	62.94 (15.12)	64.45 (14.51)	0.17
Hypertension, No. (%)	157 (72.69)	173 (80.47)	0.07
Hyperlipidemia, No. (%)	110 (50.93)	121 (56.28)	0.31
Diabetes, No. (%)	82 (37.96)	82 (38.14)	0.99
Atrial fibrillation, No. (%)	22 (10.19)	35 (16.28)	0.08
Prior stroke, No. (%)	53 (24.54)	46 (21.39)	0.51
Coronary artery disease, No. (%)	40 (18.52)	38 (17.67)	0.92
NIHSS on Arrival, mean (SD)	9.27 (6.91)	9.48 (7.3)	0.82
Thrombolytic therapy < 3 hours, No. (%)	174 (80.56)	176 (81.86)	0.82
Attempted mechanical thrombectomy, No. (%)	34 (15.74)	26 (12.09)	0.34

The mean National Institutes of Health Stroke Scale (NIHSS) score on arrival was nine for both groups, with an interquartile range (IQR) of 4-13.5 for alteplase and 4-14 for tenecteplase. The Alberta Stroke Program Early CT Score (ASPECTS) was similar in both cohorts, with a median of 10 (IQR 6.5-10) in alteplase cases versus 10 (IQR 10-10) in tenecteplase cases.

Twenty-one patients had sICH, including seven (3.2%) in the alteplase group and 14 (6.5%) in the tenecteplase group. None were on anticoagulation therapy prior to hospitalization. There were no reported blood pressure violations during the treatment course. Of these patients, three in the alteplase group and two in the tenecteplase group had mechanical thrombectomy attempted. There was no statistically significant difference in the sICH rate between the alteplase and tenecteplase groups (odds ratio: 1.44; 95% CI, 0.60-3.43; P=0.41). A higher NIHSS score on arrival was a statistically significant predictor of sICH, as shown in Table 2, based on binary logistic regression (odds ratio: 1.06; 95% CI, 1.00-1.13; P = 0.04).

**Table 2:** Logistic regression on sICH sICH: symptomatic intracranial hemorrhage; NIHSS: National Institutes of Health Stroke Scale

Symptomatic intracranial hemorrhage	Odds ratio [95% CI]
Gender	0.69 [0.29-1.65]
Hyperlipidemia	0.45 [0.17-1.16]
Atrial fibrillation	0.05 [0.03-2.3]
Prior stroke	0.62 [0.17-2.22]
Coronary artery disease	1.20 [0.35-4.05]
NIHSS	1.06 [1-1.13]
Thrombolytic therapy < 3 hours	0.32 [0.07-1.45]
Attempted mechanical thrombectomy	0.77 [0.21-2.80]
Alteplase/tenecteplase	1.44 [0.60-3.43]

There were no interaction effects or other confounding variables corrected for. Low-grade hemorrhages (HBC-1a/1b) were less common in tenecteplase-related hemorrhages (1/12, 8.33%) than alteplase-related hemorrhages (4/7, 57.14%) (Table 3).

**Table 3:** HBC in sICH HBC: Heidelberg bleeding classification; sICH: symptomatic intracranial hemorrhage; HI1: hemorrhagic infarction type 1; HI2: hemorrhagic infarction type 2; PH1: parenchymal hematoma type 1; PH2: parenchymal hematoma type 2; IVH: intraventricular hemorrhage; SAH: subarachnoid hemorrhage; SDH: subdural hematoma

HBC Class	Alteplase	Tenecteplase
1		
1a HI1	3	0
1b HI2	1	1
1c PH1	1	0
2 PH2	2	7
3		
3a PH remote	0	3
3b IVH	0	1
3c SAH	0	2
3d SDH	0	0

Heidelberg bleeding classification-2 hemorrhage was more common in tenecteplase, accounting for more than 50% of these hemorrhages. Hemorrhagic changes outside the infarcted brain tissue (HBC-3) were seen in six out of 21 patients with sICH. These six patients received tenecteplase; none were diagnosed with a large vessel occlusion, and death occurred in three out of six (50%). All patients with HBC-3 sICH were over 70 years of age, smokers, had hypertension (HTN), and did not have a known history of atrial fibrillation. Regarding stroke severity, only one patient presented with an NIHSS of three, while the remaining five presented with an NIHSS greater than 13. Our dataset showed no examples of alteplase causing HBC-3 sICH, so a logistic regression was not performed on this variable. A two-sample proportion z-test on tenecteplase produced a statistically significant increase in HBC-3 sICH over alteplase (z= -2.04, P=0.04). Additional parameters were explored for associations with other variables such as prior stroke, thrombectomy attempt, ASPECTS, NIHSS, and Modified Rankin Score (mRS). However, no statistically significant predictor was found. A detailed discussion of patients with HBC-3 hemorrhage is provided below and summarized in Table 4.

**Table 4:** HBC class 3 characteristics Y: yes; N: no; HTN: hypertension; HLD: hyperlipidemia; A-fib: atrial fibrillation; CVA: cerebrovascular accident/stroke; CAD: coronary artery disease; CHF: congestive heart failure; LKW: last known well; DTN: door to needle; ASPECTS: Alberta Stroke Program Early CT Score; ED: emergency department; NIHSS: National Institute of Health Stroke Scale; MRS: Modified Rankin Score; HBC: Heidelberg bleeding classification; N/A: not applicable

Patient	Gender	Age	Smoking	HTN	HLD	DM	A-fib	Prior CVA	CAD	CHF	Valvular Disease	NIHSS arrival	Symptoms	ASPECTS	LKW to arrival to ED (mins)	DTN mins	NIHSS discharge	MRS discharge	MRS 90 days	HBC
1	F	74	Y	Y	Y	N	N	N	N	N	N	17	Right-sided weakness, dysarthria	10	20	38	17	5	5	3a
2	F	88	Y	Y	N	N	N	N	N	N	N	19	Left-sided weakness, dysarthria	10	72	23	N/A	6	6	3a
3	F	70	Y	Y	N	N	N	N	N	N	N	3	Left-sided weakness	10	75	23	3	2	N/A	3a
4	M	92	Y	Y	N	N	N	N	Y	N	N	13	Right-sided weakness	10	27	63	N/A	6	6	3b
5	M	78	Y	Y	Y	N	N	N	N	N	N	16	Left-sided weakness	8	107	30	20	5	5	3c
6	F	83	Y	Y	N	N	N	N	N	N	N	11	Left-sided weakness	10	34	19	N/A	6	6	3c

## Discussion

Tenecteplase is a tissue plasminogen activator that offers greater specificity and a shorter infusion time as a bolus injection. Although it is not currently FDA-approved for AIS, several studies have confirmed the efficacy and safety of tenecteplase [7,10,19]. Our multicenter retrospective analysis compares the rates and patterns of sICH after administering tenecteplase versus alteplase for treating acute ischemic stroke. Our analysis demonstrated that although there was a higher number of sICH in patients given tenecteplase (14) over alteplase (seven), it was not statistically significant and could be related to the lower numbers seen in both alteplase and tenecteplase. Risk factors for hemorrhagic transformation include advanced age, history of HTN, a higher NIHSS on presentation, and the presence of atrial fibrillation [4]. Interestingly, our study showed a statistically significant association between a higher NIHSS score and the prediction of sICH. Moreover, the ASPECTS measurements were notably high in both sICH cohorts, with a median of 10.

We reviewed the imaging studies of the 21 patients with sICH, including neurovascular imaging, to exclude any vascular anomalies, potential associations with ischemic infarcts, or findings suggestive of underlying cerebral amyloid angiopathy. We observed fewer low-grade hemorrhagic transformations (HBC-1a/1b) in patients with tenecteplase-related hemorrhages, one out of 12, compared with four out of seven alteplase-related hemorrhages. These observations suggest that tenecteplase-related sICH may be qualitatively different from alteplase-related sICH. Hemorrhage outside of infarcted brain tissue, HBC-3, was seen in six out of the 21 patients with sICH, all of whom received tenecteplase. Mortality during hospitalization occurred in half of these patients with HBC-3. In most cases, a definitive mechanism was not identified to explain the hemorrhagic conversions in the patients with HBC-3. In our cohort, proposed mechanisms for these findings include underlying recent cerebral contusions and clinically silent infarcts. These lesions may help explain the relatively high ASPECTS observed in this cohort.

**Figure 1:**
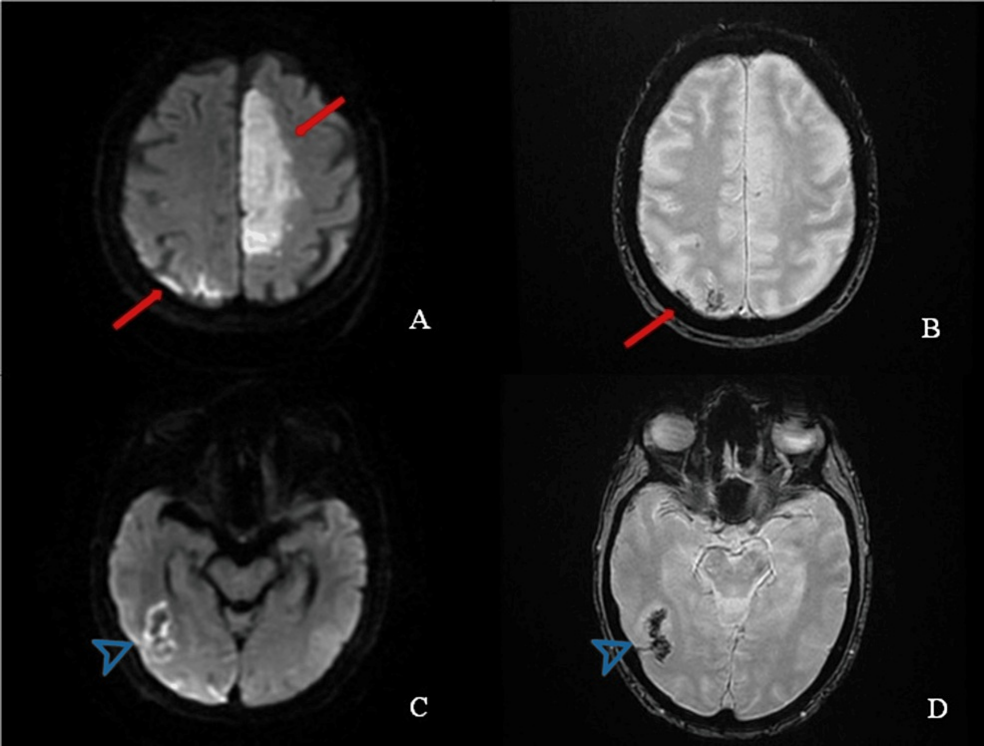
A brain MRI of the first patient with HBC-3 hemorrhagic conversion following tenecteplase administration Axial diffusion-weighted imaging (DWI) (A) and gradient recalled echo (GRE) (B) sequence MRI images of the brain demonstrate diffusion restriction in the left anterior cerebral artery (ACA) territory and right para-midline parietal lobe compatible with bilateral acute-subacute infarcts (red arrows). Axial DWI (C) and GRE (D) images of the brain at the level of the parieto-occipital lobe demonstrate diffusion restriction and susceptibility artifacts in the right para-midline parietal and occipital lobes compatible with intraparenchymal hemorrhage (arrowhead). HBC: Heidelberg bleeding classification

As shown in Table 4, the first patient with hemorrhagic conversion, HBC-3 type, had a recent fall four days before stroke presentation to the ED, which resulted in a broken arm and shoulder dislocation, but without reported radiographic or clinical signs of head trauma. The patient had an NIHSS of 17 and presented with aphasia and right-sided weakness. An MRI of the brain after tenecteplase administration demonstrated right posterior parietal lobe hemorrhage with contralateral left anterior cerebral artery (ACA) AIS (Figure 1).

The third patient was found down after a presumptive fall, with no signs of trauma on the secondary trauma survey. The patient presented with an NIHSS of three and left-sided weakness, for which tenecteplase was administered. The MRI demonstrated a right middle cerebral artery (MCA) AIS with hemorrhage in the ischemic bed and parenchymal hemorrhage in the right occipital region and left MCA territory. The areas of post-tenecteplase hemorrhage in the above two patients were located outside of the ischemic bed, and a prominent similarity between the two patients is a history of trauma without an evident head injury. This presents the possibility of underlying cerebral contusions that may have predisposed them to hemorrhagic transformations in these anatomic locations. Additionally, while no clear history of atrial fibrillation was present for the first and third patients, potential embolic bilateral infarctions with hemorrhagic transformation in some areas cannot be excluded, as pre-tenecteplase head CT on the first patient demonstrated a chronic left frontal stroke. However, these patients did not exhibit apparent localizing signs of bilateral strokes on initial examination in the ED (Table 4). The AHA considers "severe head trauma" within three months before AIS as a contraindication for IV thrombolytic therapy, and therapy should be "carefully considered" in major trauma not involving the head within 14 days before AIS [21,30].

**Figure 2:**
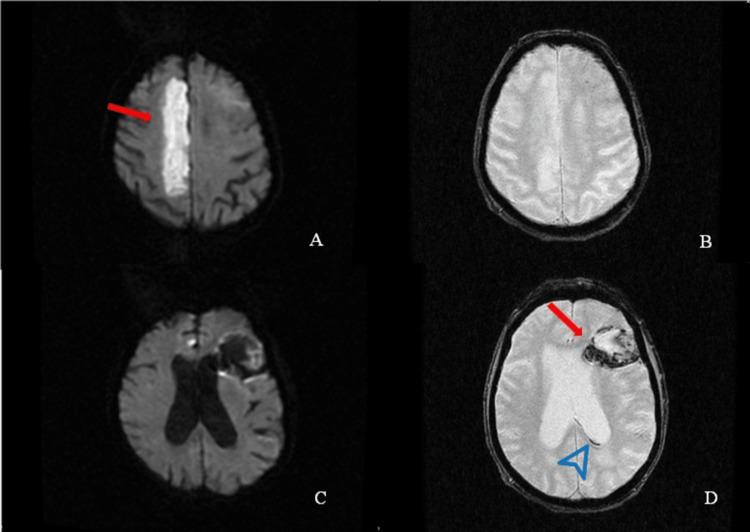
A brain MRI of the second patient with HBC-3 hemorrhagic conversion following tenecteplase administration Axial diffusion-weighted imaging (DWI) (A) and gradient recalled echo (GRE) MRI images of the brain at the level of the centrum semiovale demonstrate diffusion restriction (arrow) compatible with acute-subacute infarct in the right ACA territory with no hemorrhage in the corresponding territory on the GRE sequence. Axial DWI (C) and GRE (D) sequence images of the brain demonstrate left frontal middle cerebral artery (MCA) territory acute hemorrhagic infarct (arrow), with trace left intraventricular hemorrhage (arrowhead). HBC: Heidelberg bleeding classification

The second patient presented to the ED for left-sided weakness and slurred speech with an NIHSS of 19. The brain MRI demonstrated right ACA AIS with acute hemorrhage in the left frontal MCA territory and a trace left intraventricular region (Figure 2).

A review of vascular imaging ruled out azygos ACA, which makes it unlikely that a single embolism resulted in bilateral ACA territory infarctions and suggests another mechanism for this patient’s hemorrhagic pattern.

The fourth patient presented to the ED after a syncopal event at dinner without signs of head trauma. The neurological exam was significant for right-sided weakness and an NIHSS of 13. The post-tenecteplase MRI revealed left ACA/MCA watershed infarcts in the setting of greater than 70% left cervical internal carotid artery (ICA) stenosis. Additionally, there was evidence of acute parenchymal hemorrhage in the left temporal lobe and bilateral intraventricular hemorrhage (IVH). The fifth patient presented with the right MCA symptoms and an NIHSS of 16. The post-tenecteplase MRI brain demonstrated acute right MCA AIS, left occipital and right temporal lobe infarcts, and bilateral occipital subarachnoid hemorrhage (SAH). The sixth patient presented with left hemiparesis and an NIHSS of 11. The post-tenecteplase CT brain demonstrated diffuse SAH and IVH. The etiology of SAH was most likely secondary to spinal cervical arteriovenous malformation (AVM), only diagnosed on post-thrombolytic MRA neck and questionably on a retrospective review of pre-tenecteplase CTA neck. Spinal hemorrhagic disorders are rare, especially as an acute ischemic stroke presentation or mimic [31,32]. There is no literature report of spinal AVM rupture after thrombolytic therapy. The patient had no reported neck pain or bilateral neurological symptoms. An unruptured cerebral or spinal AVM is not a contraindication for thrombolysis administration.

After reviewing the current literature, there appears to be somewhat conflicting data regarding the safety and efficacy of tenecteplase compared to alteplase. A recent meta-analysis and multiple multicenter randomized trials reported that tenecteplase in AIS was non-inferior to alteplase in AIS with comparable safety and effectiveness [10,11,14]. Similarly, a systematic review of multiple non-randomized studies showed a comparable rate of ICH, better recanalization, and earlier neurologic improvement with tenecteplase compared to alteplase [33]. Others showed a trend toward an even lower rate of ICH with tenecteplase patients [18]. As of early 2023, The Comparative Effectiveness of Routine Tenecteplase vs. Alteplase in Acute Ischemic Stroke (CERTAIN) Collaboration reported real-world experience from more than 100 hospitals in New Zealand, Australia, and the US. This study reported lower rates of sICH in patients treated with 0.25 mg/kg tenecteplase than with alteplase (1.8% vs. 3.6% for alteplase; P<.001) [25]. A recent New Zealand Central Stroke Region report also showed lower sICH, better 3-month functional outcomes, and shorter onset to needle times [34]. Conversely, a retrospective study that included 30,643 patients in the United States found that the occurrence of sICH was higher in non-mechanical thrombectomy AIS candidates who were treated with tenecteplase compared to alteplase (7.9% versus 5.1%, P<0.001), which aligns more with our findings [24].

Our study has several limitations, including a small patient population, retrospective cohort design, and a relatively narrow time frame since the implementation of tenecteplase for AIS across the hospital system. Other limitations include the inability to account for uncontrolled confounders, secular trends, institutional learning curves, and the Hawthorne effect due to more close monitoring of ICH. Including the full spectrum of HBC classes limits direct comparison to other studies. Previous literature suggested that HBC-1a and 1b after administration of alteplase were not linked to worse outcomes [35]. However, five hemorrhagic transformation cases in our study were symptomatic in this class. One of the strengths of our study is that the sICH scans after thrombolytics were reviewed by vascular and neuroendovascular physicians who were not part of the patient treatment team. Additionally, there were patient-level data that were reviewed for the patients specifically with sICH. This study describes the bleeding patterns associated with tenecteplase administration, which is lacking in the existing literature. These findings warrant further investigation in other cohorts to gain more insights.

## Conclusions

Compared with alteplase, our study revealed a higher rate of sICH with tenecteplase that was not statistically significant and a higher rate of HBC-3 hemorrhages that was statistically significant. The proposed mechanism of bleeding is hemorrhagic conversion in clinically silent infarcts and contusions underlying the lesions. Further studies are needed to confirm our findings and determine predictive risk factors.
